# Editorial: Survival in Extreme Environments – Adaptation or Decompensation?, Volume I

**DOI:** 10.3389/fphys.2022.836210

**Published:** 2022-02-04

**Authors:** Torkjel Tveita, Ingrid Eftedal, Sanjoy Deb

**Affiliations:** ^1^Anesthesia and Critical Care Research Group, Department of Clinical Medicine, UiT, The Arctic University of Norway, Tromsø, Norway; ^2^Division of Surgical Medicine and Intensive Care, University Hospital of North Norway, Tromsø, Norway; ^3^Department of Circulation and Medical Imaging, Faculty of Medicine and Health Sciences, NTNU Norwegian University of Science and Technology, Trondheim, Norway; ^4^Faculty of Nursing and Health Sciences, Nord University, Bodø, Norway; ^5^Centre for Nutraceuticals, School of Life Sciences, University of Westminster, London, United Kingdom

**Keywords:** diving, hunting, fishing, extraction work, farming

The history of human migration is also the history of successful biological interactions with new environments, where maintenance of body homeostasis is the fundamental property. Equilibrium of body homeostasis is the product of many regulatory physiologic mechanisms and seems to result from a natural resistance to change from a pre-existing optimal biological condition. Chronic or functional adaptation is the product of processes which may run for the whole life of an individual. Human adaptive processes may take thousands of years, like the process of standing and walking erect or adaptation to living at a high altitude which is possible due to some adaptation, but these ongoing processes need much more time to be optimal. However, the focus of the Research Topic is related to acute adaptation to a new equilibrium which may take place within seconds. Challenges to maintain biological equilibrium in a new environment, or more simply, to stay alive, include climate, ambient temperature and pressure, high altitude, water immersion, and the immediate access to naturegiven resources like food, water, and shelter. When humans are exposed to extreme environments, accidents may occur; the victims are no longer adapted and may decompensate due to hyper/hypothermia, oxygen deprivation, high-/low ambient pressures, or drowning. Professional workers like divers, extraction industry workers, anglers and hunters, seamen, or people seeking these elements during leisure or recreational activity, all depend on their ability to adapt, but share the same potential threat that their biological homeostasis will disrupt in case of an accident. The rescuers, on the other hand, may often meet other challenges, like lack of practice to institute proper care of patients with these relatively rare conditions, or worse, attempting to treat such decompensated patients where the guidelines for treatment are not yet written. To write these guidelines, we are in urgent need of new knowledge. Due to obvious reasons, some of this new knowledge of complex pathophysiologic mechanisms evoked by accidents can only be collected from animal models experiments. However, novel techniques allow us to conduct clinical studies on subjects during their daily activities under harsh working conditions. Due to the very intrinsic integrative nature of the physiological mechanisms involved in adaptation—or not—to extreme environmental challenges, it is sometimes difficult to understand them in their entirety. Fortunately, new opportunities have arisen with the advent of biomolecular techniques, providing tools to better understand the interactions between the different levels of integration within the body, allowing us to see the bigger picture.

In this Research Topic we present a valuable collection of new preclinical and clinical research data with the potential as background information to broaden our insight into how to improve our physiologic adaptation, or at least to be better prepared when meeting a challenging environment. We have collected data with translational potential to better manage critical care conditions after exposure to such environments.

Anecdotal information exists on how to face and survive harsh environments. Those living by the north Atlantic coastline learned early from offshore fishermen that to stay alive out there you must keep warm and get enough rest. The message was that becoming chilly or sleepless may be as dangerous as consuming alcohol due to the potential threat of losing mental control. Aspects of this information are elegantly taken into the research lab and presented by Færevik et al. in the Research Topic. They showed that test persons, adequately dressed for an ambient temperature of −2°C, limited to 6 h exposure time during the night after a good night sleep before that, remained mentally alert and warm. Likewise, over the past 60 years, an understanding exists that victims of accidental hypothermia may survive even if rewarming is started after prolonged periods of cardiac arrest, and the saying “Nobody is dead until warm and dead” has become a slogan. The main reason is that over the same years the medical health care system, from prehospital care to in hospital advanced medical care, together have managed to reduce morality of accidental hypothermia from 52 to 80% at the beginning to the present 28–35%. However, this success rate is closely linked to accidental hypothermia patients without cardiac arrest, whereas survival rate of patients in cardiac arrest is much lower. The recommended treatment of hypothermic cardiac arrest is rapid patient transfer under continuous cardiopulmonary resuscitation (CPR) to a hospital equipped for in-hospital rewarming using extracorporeal membrane oxygenation (ECMO). But sometimes, mostly due to weather conditions, patient transport to a hospital with ECMO might be impossible and the search for alternative methods to ECMO rewarming must be investigated. In the preclinical experimental work by Nivfors et al. an alternative method, warming by continuous perfusion of the pleural cavity with warm water (Closed thoracic lavage) was tested out following 3 h of continuous CPR. This work demonstrated the positive effect of CPR at reduced core temperature (27°C) to maintain organ perfusion, but also demonstrated the need for ECMO rewarming as thoracic lavage failed to establish a perfusing rhythm during rewarming. The work by Mohyuddin et al. demonstrated two facts; the hemodynamic stability of an intact experimental model with spontaneous circulation to undergo cooling and rewarming, as well as the positive effects of the adrenergic agent epinephrine, to support cardiovascular function during hypothermia. This work may also underline the clinically recognized differences in survival between patients with cardiac arrest vs. maintained spontaneous circulation. When rewarming patients with maintained spontaneous circulation they regularly have an increase in total vascular resistance. In addition, they may suffer from may suffer from a varying degree of cardiac failure. This calls for immediate pharmacologic interventions for circulatory support, but a lack of consensus of pharmacologic strategy in hypothermic patients exists. In principle, pharmacologic interventions during acute cardiac failure contain two elements: vasodilation or myocardial support, or their combination. We saw that the cardiac supportive effects of epinephrine were also maintained at low temperatures, but the pharmacologic effects of vasodilating substances during hypothermia are so far not well-described. The work by Lund Selli et al. shows that the vasodilator effects of the phosphodiesterase 5 (PDE5) inhibitor, sildenafil, to inhibit the elimination of cGMP, to maintains vascular smooth muscle activity (vasodilation), is maintained during *in vitro* cooling to 20°C.

Cold-water divers also experience hypothermia, and those who are cold during the ascent from diving may be at increased risk of decompression sickness (DCS). DCS is a multifaceted disease with a wide range of symptoms and contributors. Gaustad et al. used a temperaturecontrolled rodent model to target effects of a 2°C drop in core temperature during decompression on post-dive vascular bubble formation and hemodynamic function. They found no changes in bubble formation to substantiate increased DCS risk. However, cardiac output and stroke volume fell after the dive, possibly because of reduced left ventricular preload secondary to increased pulmonary resistance. Other have found reduced cardiac output to be associated with increased muscular sympathetic nerve activity, but as there was no post-dive increase in total peripheral vascular resistance in Gaustad's study this explanation appears less likely. Whether the observed outcome was triggered by undetected bubbles remains to be explored. Rodents are also useful preclinical models for pharmacological interventions aimed at alleviating injury to divers. Zhou et al. reported a favorable outcome of normobaric oxygen (NBO) in combination with inhibition of mitogenactivated protein kinase MEK1/2 on DCS in rats with spinal cord injury. They found that the combination resulted in lower incidence of DCS compared to that of animals treated with NBO or MAK1/2 inhibitor alone, and propose that the effect is attributable to increased expression of heat shock protein HSP32.

As useful as animal models are for understanding of physiology and pathology, clinical medicine still depends on studies of human subjects. But controlled prospective studies of human maladaptation are difficult. Provoking potentially life-threatening disease in healthy subjects is for obvious reasons not possible, and the variety in symptoms and severity associated with DCS complicates the Research Topic. Magri et al. overcame this in a study of immune and inflammatory changes in divers with DCS. The detailed insight of the molecular ethiology of DCS provides possible starting points for the search for biomarkers and drugable targets for prevention and improved treatment.

Saturation diving is an extreme occupation where individuals are exposed to a high-pressure hyperoxic environment for prolonged periods. Individuals must adapt to physiological stress to maintain health and physiological function. Monnoyer et al. demonstrated that a 4-weeks in this environment during a commercial saturation dive at 200 m resulted in transient alterations to the oral microbiome. This oral microbiome offers some fascinating insight into both oral and systemic health and disease states, with imbalances in the oral microbial ecosystem may manifest in disturbances to health. This investigation found transient changes in oral bacterial diversity and abundance following decompression. These changes were maintained throughout the bottom phase of the dive and appeared to return to pre-dive levels on return to the surface. This attenuation of anaerobic activity during the bottom phase may contribute to a saturation diver's health outcome. Several factors determine the health and wellbeing during a saturation. An area of notable interest is the role of the diet. Deb et al. present the first analysis of energy expenditure during a commercial saturation dive. Several interesting observations were presented, which may have significant practical implications. It was evident that divers were in a substantial negative energy balance during the 10-day measurement period, with overall energy expenditure being positively correlated with the time spent working underwater. It is apparent that there is an intrinsic link between diet, occupational work and the environmental exposure of saturation diving. These investigations provide insight into the adaptive physiological responses to commercial saturation diving and present interesting avenues to improve the health and wellbeing of occupational divers.

Taken together, studies in this Research Topic present consequences of exposure to extreme environments, and potentional effects of adaptational interventions. This new information on the consequences may support the development of updated guidelines intended to improve safety and survival.

## Author Contributions

All authors listed have made a substantial, direct, and intellectual contribution to the work and approved it for publication.

## Conflict of Interest

The authors declare that the research was conducted in the absence of any commercial or financial relationships that could be construed as a potential conflict of interest.

## Publisher's Note

All claims expressed in this article are solely those of the authors and do not necessarily represent those of their affiliated organizations, or those of the publisher, the editors and the reviewers. Any product that may be evaluated in this article, or claim that may be made by its manufacturer, is not guaranteed or endorsed by the publisher.

